# Impact of sonication power on the degradation of paracetamol under single- and dual-frequency ultrasound

**DOI:** 10.1016/j.ultsonch.2023.106564

**Published:** 2023-08-19

**Authors:** Mehrdad Zare, Madeleine J. Bussemaker, Efraím A. Serna-Galvis, Ricardo A. Torres-Palma, Judy Lee

**Affiliations:** a School of Chemistry and Chemical Engineering, University of Surrey, Guildford GU2 7XH, United Kingdom; b Grupo de Investigación en Remediación Ambiental y Biocatálisis (GIRAB), Instituto de Química, Facultad de Ciencias Exactas y Naturales, Universidad de Antioquia UdeA, Calle 70 # 52-21, Medellín, Colombia; c Catalizadores y Adsorbentes (CATALAD), Instituto de Química, Facultad de Ciencias Exactas y Naturales, Universidad de Antioquia UdeA, Calle 70 # 52-21, Medellín, Colombia

**Keywords:** Pharmaceuticals sonodegradation, Power effects, Cavitation, Dual-frequency ultrasound, Dosimetry, Sonoluminescence, Sonochemiluminescence

## Abstract

The effects of sonication power on the ultrasonic cavitation and sonochemistry as well as the degradation of paracetamol were studied and compared for single- and dual-frequency sonoreactors. For the single-frequency sonication, a 500 kHz plate transducer was employed, with three different calorimetric powers of 8.4, 16.7 and 27.9±3.9 W. For the dual-frequency sonication, the plate transducer was perpendicularly coupled with a low-frequency 20 kHz ultrasonic horn, and three calorimetric powers of 27.9, 33.4, 44.6±3.9 W were studied. At all the studied powers, dual-frequency sonication led to a synergistic effect in the degradation of paracetamol, though varying the power of the horn did not affect the degradation rate. A comparison of the degradation data versus the yield of oxidants as well as the overall intensities of sonoluminescence and sonochemiluminescence suggested the degradation is by the action of oxidants near the surface of the bubbles as the major reaction mechanism. Despite the enhancement observed for the degradation, dual-frequency sonication had no significant effect on the yield of either of the oxidants, regardless of the applied power to the horn. In contrast, dual-frequency sonication decreased the overall sonoluminescence and sonochemiluminescence intensities at all powers studied, suggesting that the application of dual-frequency sonication reduces the size of cavitation bubbles. Normal distribution function analysis confirmed dual-frequency sonication resulted in smaller sonoluminescing bubbles, hence the reduction in the sonoluminescence intensity. The increase in degradation rate under DFUS is attributed to the increase in the transfer of paracetamol from the bulk towards the bubbles. As a result, the availability of the pollutant molecules in the vicinity of the bubbles to react with HO• would increase and consequently, the degradation rate would enhance under DFUS.

## Highlights

• Three powers for either of the frequencies were studied under SFUS (500 kHz) and DFUS (500+20 kHz).
• Oxidation by sono-generated ROS was the degradation mechanism under SFUS and DFUS.
• DFUS at all powers led to a synergistic effect while having no significant effect on ROS yield.
• DFUS slightly reduced SL by reducing the bubble size distribution.
• DFUS enhanced degradation by enhancing mass transfer via stabilising the cavitation bubbles.

## 1 Introduction

In recent years, serious concerns about contaminants of emerging concern (CEC) [1], in particular, pharmaceuticals [2], [3], have been raised. Studies have shown that these compounds have direct and indirect adverse effects on the environment and human health [3], [4], ranging from endocrine and reproduction cycles to gene expressions of living creatures [5], [6]. Among various CEC pharmaceuticals, analgesics including paracetamol (PCM) have one of the highest measured environmental concentrations [7]. PCM is an over-the-counter (OTC) medicine with unregulated consumption that can cause liver and kidney impairment by producing hepatoxic metabolites like N-acetyl-p-benzoquinone imine [8], [9] Due to the inadequacy of the current municipal water treatment plants [10], [11], [12], studies have been conducted in pursuit of alternative methods [13]. Among the alternatives to deal with pharmaceuticals, sonodegradation as a green advanced oxidation process (AOP), which does not require the addition of chemicals, is promising [14].

Sonodegradation is based on the severe implosion of the cavitation bubbles formed during the sonication of an aqueous solution, which results in a transient pressure and temperature rise (∼5000 K and ∼1000 bar) in the bubbles’ cores [15], [16]. These severe conditions lead to the formation of nonequilibrium plasma inside the bubbles [17] and cause the water and oxygen molecules inside the bubbles to break and form reactive oxidative species (ROS) such as HO•, that can degrade organic compounds at the interface and to some extent in the bulk solution [15], [18]. Also, the severe collapse condition of the cavitation bubbles might result in the pyrolysis of the volatile organic compounds that can enter the cavitation bubbles [19], [20]. Some hydrophobic pollutant molecules around the bubbles could also enter the bubbles by the nanodroplet injection during the implosion [21] and be degraded via pyrolysis.

Sonodegradation efficiency has been enhanced by optimising the sonication conditions [22], [23], and combining sonodegradation with other AOPs to take advantage of any possible synergy [24], [25]. A portion of these studies has investigated the combination of multiple ultrasound frequencies, reporting a synergistic effect [26], [27], [28], [29], [30], [31], [32], [33], [34]. Including one low-frequency ultrasound (<100 kHz) in multi-frequency irradiation is beneficial in terms of sonochemistry because of the low cavitation threshold at low frequencies [27], [28], [32], [33], [34], [35], [36], [37]. Also, Lee and Oh [28] have shown that the synergistic effect from dual frequency systems could be maximized if the transducers are arranged in an opposing configuration (perpendicularly) when a high-frequency ultrasound is involved with a low to moderate power. The synergistic index reported in the literature ranges from above 1 [29], [34] to above 3 [27] and in some cases, antagonistic results are observed [38], [39] where the simultaneous application of multiple frequencies diminished the sonochemical activity. The observed synergy is attributed to the magnification of the nonlinear oscillation of cavitation bubbles under the combined and simultaneous resonances of the input frequencies [40]. Nevertheless, the theory cannot explain all the various synergistic effects reported or in some cases, the antagonistic results reported in the literature for similar sonication conditions [41]. Therefore, the true mechanism behind the synergy is still unclear and there is a need to further study multi-frequency sonoreactors to close the knowledge gap on the enhancement observed in multi-frequency sonodegradation of CEC.

Zare et al.[42] investigated the sonochemical activity and degradation of PCM under a wide range of frequencies (20–2000 kHz) in single- and dual-sonication modes and attributed the main PCM degradation mechanism to chemical oxidation by ROS, in particular, HO•. Also, applying dual-frequency ultrasound (DFUS) resulted in synergy in the degradation of the PCM, despite no synergy being observed for the ROS yield. Due to the lack of enhancement in ROS yield, the synergy was attributed to the enhancement in mass transfer caused by the introduction of the second frequency to the acoustic field. However, the effect of DFUS sonication parameters such as sonication power on this enhancement and the consequent synergy remains unclear.

Although some studies [27], [28], [32], [33] have investigated the effect of sonication power under DFUS, there is still a lack of a comprehensive study, clarifying how the power of either of the frequencies could affect the sonochemistry as well as the synergy under DFUS. In the studies by Feng et al. [27], Zhao et al. [32], and Zhu et al. [33] only the power of one of the applied frequencies was varied and the power of the other frequency was kept constant. In contrast, Lee and Oh [28] studied three power levels but the same power was applied to both frequencies, making it difficult to conclude if a particular frequency was more sensitive to power. To address this research gap, three different powers will be applied to each frequency under single- and dual-frequency sonication in a full-factorial mode, and its cavitation activity will be fully characterised via ROS dosimetry, SL and SCL measurements. Also, the degradation of a representative CEC will be investigated and compared. Based on a previous study [42], 500 kHz ultrasound was selected as the suitable frequency to be used as single-frequency ultrasound (SFUS) and to be paired in an opposing configuration with a 20 kHz ultrasound to give DFUS. For the degradation studies, PCM was selected as the target pharmaceutical, which is widely found in water/wastewater [43].

## 2 Materials and methods

### 2.1 Chemicals

Paracetamol (PCM, 4-Acetaminophenol, purity ≥98%) was purchased from ACROS Organics and used as received. For the iodide dosimetry experiments, potassium iodide (KI, purity ≥99.5%) and ammonium molybdate tetrahydrate ((NH_4_)_6_Mo_7_O_24,_ purity ≥99%) were obtained from Fisher Chemicals and Sigma-Aldrich, respectively. The sodium hydroxide (NaOH, purity ≥98%), and luminol (5-Amino-2,3-dihydro-1,4-phthalazinedione, purity ≥97%) used for sonochemiluminescence (SCL) measurements were obtained from Sigma-Aldrich. All solutions were prepared using Milli-Q water (18.2 MΩ).

### 2.2 Sonoreactor setup

**Fig. 1:**
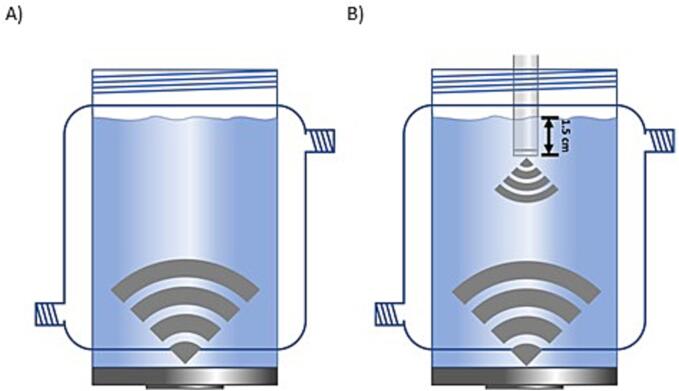
The schematic view of the sonoreactor setup in A) SFUS and B) DFUS mode of operation.

A cylindrical jacketed glass reactor with an internal diameter of 6.7 cm and a capacity of 400 mL was used, with a 500 kHz plate transducer (the high-frequency ultrasound) mounted at the bottom for single-frequency ultrasound (SFUS) irradiation. The plate transducer was supplied by Honda Electronics comprising a 5 cm in diameter piezo-electric ceramic attached to a 10 cm in diameter vibration plate. For dual-frequency ultrasound (DFUS), a 20 kHz ultrasonic horn (Fisherbrand™ Q700 Sonicator) as the low-frequency US was paired with the high-frequency US in an opposing configuration. In all the experiments, the horn tip was dipped 1.5 cm into the solution (Fig. 1) which is consistent with our previous study [42]. The horn position was chosen to avoid disturbing the liquid surface too much while maximising the volume between the horn and plate transducer.

To study and compare the effects of the sonication power under SFUS and DFUS, three different power levels were applied for either of the frequencies i.e., the SFUS experiments were conducted under three different power levels, and a full-factorial matrix of 9 different power combinations was applied for DFUS experiments (Table 1). Accordingly, for the SFUS and DFUS experiments, amplifier load powers of 10, 20, and 30 W were applied to the plate transducer, delivering the calorimetric power of 8.4, 16.7, and 27.9±3.9 W to the solution (corresponding to the power densities of 20.9, 41.8, and 69.6±9.8 W.L^−1^, respectively). Also, for the DFUS experiments, the amplitudes of 20, 30, and 40% of maximum were applied to the ultrasonic horn, which alone resulted in the calorimetric powers of 27.9, 33.4, and 44.6±3.9 W (corresponding to the power densities of 69.6, 83.6, and 111.4±9.8 W.L^−1^, respectively). It should be noted that under each DFUS power condition, the total calorimetric power exerted on the liquid is the sum of the calorimetric power of the plate transducer and the ultrasonic horn. Table 1 summarises the calorimetric powers applied for the power studies.

**Table 1:** The total calorimetric powers applied for the SFUS and DFUS experiments, to study the effect of sonication power. Due to the errors accompanied by the employed measurement instruments such as the thermometer, all the values reported in the table have an error of 3.9 W.

		SFUS		DFUS		
				US Horn Amplitude (%)		
				20 (27.9 W)	30 (33.4 W)	40 (44.6 W)
						
Plate Transducer Load Power (W)	**10**	8.4		36.2	41.8	52.9
	**20**	16.7		44.6	50.1	61.3
	**30**	27.9		55.7	61.3	72.4
						

For all of the experiments, the initial temperature of the solution was set to 16 °C before starting the sonication and was maintained during the experiment, using a chiller (Julabo FL300) within 5 °C above the initial temperature.

### 2.3 Experimental and analytical methods

#### 2.3.1 Degradation of paracetamol

For each power combination under SFUS and DFUS experiment, 400 mL of fresh paracetamol solution with a concentration of 5 mg.L^−1^ was sonicated for 60 min. The degradation of PCM was monitored using an Evolution 201 by Thermo Scientific UV–Visible spectrophotometer (UV–Vis.) at 243 nm [44] (Fig. S1 of the supplementary data), by analysing 2 mL samples every 10 min. It should be noted that this method quantifies the degradation of not only PCM but also the sonodegradation intermediates that share UV absorption with PCM at the applied wavelength [42]. After the measurements, the samples returned to the solution to maintain the power density exerted on the solution. The sonication was on throughout the experiment, the UV–Vis. measurements and returning the sample to the reactor did not take longer than 30 sec.

#### 2.3.2 ROS production

The yields of the main ROS (HO• and H_2_O_2_) produced by the sonication were determined using two sets of dosimetry experiments. For the yield of HO•, 400 mL of 0.1 M KI fresh solution underwent the same sonication conditions as for degradation experiments, and the HO• concentration was measured by taking 2 mL samples at different times (1,2,3,5,7 and 10 min after starting the sonication). The generation of HO• is a zero-order reaction [28], [45], [46], [47] i.e., the concentration shows linear dependency only to the sonication time with a constant yield (slope). Therefore, the KI solution was sonicated for only 10 min to generate enough data points to determine the yield. As for the degradation experiments, the samples were returned to the reactor after the analysis to maintain the power density exerted on the solution. The sonication was on throughout the experiment and UV–Vis. measurements and returning the sample to the reactor did not take longer than 30 s.

The HO• produced during the sonication oxidises the iodide (I^−^) in the solution via Reactions (1) to (4), yielding I_3_^−^ [35]:

(1) $$HO\cdot \;+\;I^{-}\to \;{\mathrm{OH}}^{-}+I\cdot$$

(2) $$I\cdot \;+\;I^{-}\to \;I_{2}^{-}$$

(3) $${2\;I}_{2}^{-}\;\to \;I_{2}+\;2\;I^{-}$$

(4) $$I_{2}+\;I^{-}\to I_{3}^{-}$$

Considering the direct proportionality of the concentration of I_3_^−^ with that of HO•, the yield of the oxidant was calculated by measuring the concentration of I_3_^−^ based on the Beer-Lambert Law, using an Evolution 201 by Thermo Scientific UV–Visible spectrophotometer at the wavelength of 350 nm and with a molar absorptivity (ε) of 26,000 L⋅mol^−1^⋅cm^−1^ for a path length of 1 cm [48].

Using this method, the yield of H_2_O_2_ produced due to the recombination of HO• cannot be accounted for since it does not react instantly with the iodide in the KI solution [48], [49], [50]. Hence, a second set of dosimetry experiments was conducted by adding 0.5 mM ammonium molybdate to the 0.1 M KI solution. The ammonium molybdate within the KI solution acts as a catalyst for Reaction (5) [48]:

(5) $$H_{2}O_{2}\;+\;2I^{-}\to \;{2\;OH}^{-}+I_{2}$$

The I_2_ produced via Reaction (5), as well as Reaction (3), then undergoes Reaction (4), giving the yield of total ROS (both the HO• and H_2_O_2_) simultaneously. The yield of H_2_O_2_ could be achieved by subtracting the HO• yield, measured through the first set of dosimetry experiments, from the yield of total ROS. It is worth noting that the H_2_O_2_ yields achieved using this method have been verified [42] using the method explained by Alegria et al. [49].

All the experiments were repeated at least three times, and the average, as well as the standard deviation of the measurements, were presented in this work unless otherwise stated.

#### 2.3.3 Sono- and sonochemi-luminescence intensity

To better understand and compare the impact of the sonication power on the acoustic field under SFUS and DFUS, the spatial distribution and intensities of the SL and SCL were captured using an ANDOR iXon3 EMCCD camera, operating at −70 °C. To eliminate the noise by the ambient light, the photography was conducted within a lightproof box. Also, background photographs were recorded before and after the sonication, and the average of the light intensities recorded in the two background photographs was subtracted from the main photograph to yield the SL and SCL emission.

For SL measurements, 400 mL of Milli-Q water was photographed under sonication with the same power combinations applied for the degradation experiments. The camera settings used were 20 s for the exposure time and 100 for the Electron Multiplying (EM) Gain.

For the SCL measurements, 400 mL of 0.1 M NaOH solution containing 1 mM luminol was sonicated under the same power combinations. The photographs were taken using the same procedure mentioned above for the SL measurement, but because luminol generates a considerably higher brightness compared to SL, a shorter exposure time of 4 s and a lower EM gain level of 4 was used to obtain a clear image.

The light intensities recorded by the individual camera pixels were also analysed statistically by calculating the normal distribution function (NDF) for their count of occurrence. For this purpose, pixels with intensities less than 400 a.u. were filtered out as noise. After calculating the arithmetic mean and the standard deviation of the remaining data, the normal distribution function was calculated using Microsoft Excel (v2303) based on the values as well as the abundance of each data point.

## 3 Results

In the following, the results of the power studies for the degradation rate of PCM and the UV-absorbing intermediates [42], the ROS dosimetry, as well as SL and SCL, are presented. The graphs in this section present the measurement results for each sonication power mentioned in Table 1, first with respect to the power of the ultrasonic horn (the rows of Table 1) and then concerning the power of the plate transducer (the columns of Table 1). For easier comparison, all the values shown in the graphs are normalised based on the corresponding experimental data of the DFUS with the driving power combination of 20 W and 30% of maximum amplitude (Table S2 of the supplementary data), for the plate transducer (500 kHz) and the horn (20 kHz) frequency US, respectively.

### 3.1 Degradation of paracetamol

Sonodegrataion of organic pollutants is a heterogeneous reaction the kinetics and the rate order of which could be dependent on the transport of the pollutant molecules towards the cavitation bubbles. To accurately determine the reaction rate order, the pseudo-1st- and pseudo-2nd-order reaction kinetic models were applied to the PCM degradation data and statistically compared (Table S1 of the supplementary data). The comparison revealed that the pseud-2nd-order model shows better compliance with the data. This is also supported by the literature [42], [51] where the degradation reaction follows the pseudo-2nd-order rate at low concentrations, and by facilitating the transport via increasing the bulk concentration the reaction rate switches to the pseudo-1st-order model.

**Fig. 2:**
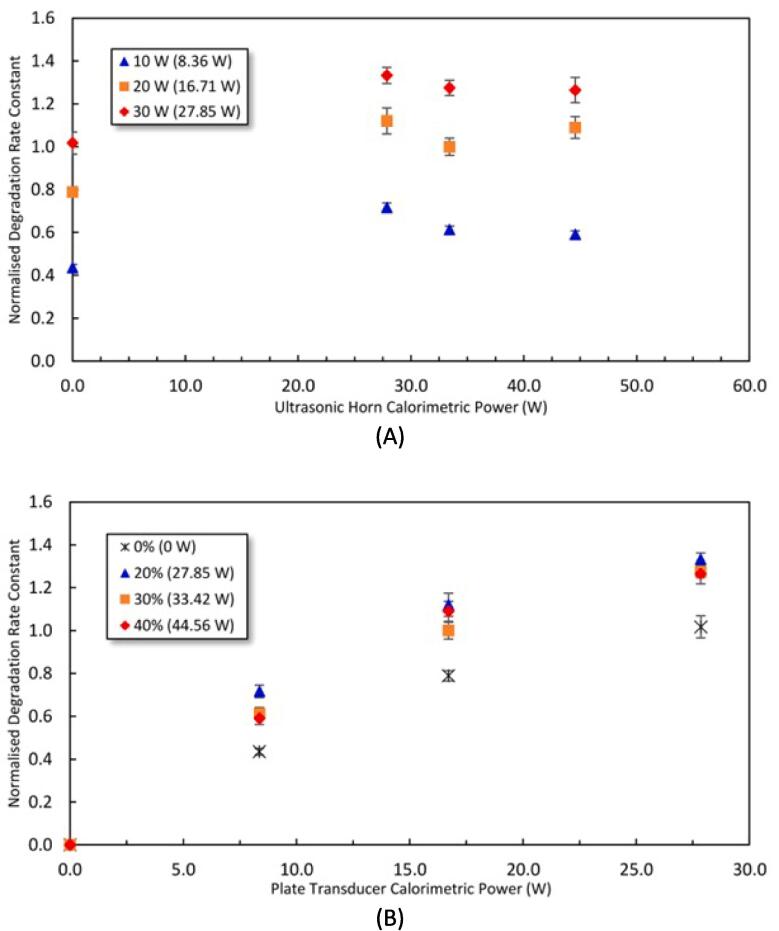
Normalised PCM degradation rate constant as a function of calorimetric power of A) the ultrasonic horn and B) the plate transducer. The results presented at 0 W in the figures are for the SFUS of either of the frequencies in the absence of the other frequency. The legend shows the driving power for the plate transducer in (A) and the horn in (B) where the corresponding calorimetric powers are mentioned in brackets. The degradation rate constants are normalised against the degradation rate constant of 6.30×10^−4^±7.34×10^−5^ obtained under DFUS (500 kHz at 20 W and 30% for the 20 kHz horn).

Fig. 2 shows the effect of the sonication power on the degradation of PCM as a function of calorimetric power for the horn (Fig. 2A) and the plate transducer (Fig. 2B). In both figures, DFUS gave a higher degradation rate compared to SFUS. However, for all of the plate transducer’s power settings, increasing the horn power from 20% to 40% did not further enhance the PCM degradation rate (Fig. 2A). It should be noted that applying the horn alone at its studied powers resulted in no detectable PCM degradation. According to Son et al. [52], this could be due to the horn tip not being immersed deep enough in the solution and will need to be investigated in the future work. In comparison, the normalised degradation rate shows a significant linear increase with increasing plate transducer’s power (Fig. 2B). In general, of all the power combinations studied, the optimum power combination in terms of PCM degradation is at maximum plate transducer power (30 W) and lowest ultrasonic horn power (20%).

**Fig. 3:**
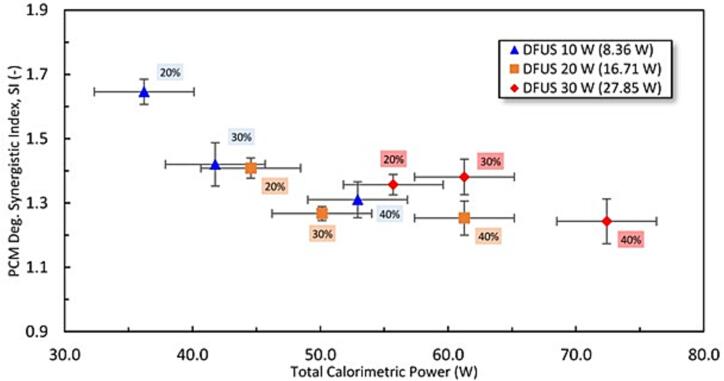
The PCM degradation SI under DFUS versus the total calorimetric power applied. The legend shows the driving power for the plate transducer where the corresponding calorimetric powers are mentioned in brackets. The driving amplitude % for the horn is labelled next to the data.

The enhancement observed in the PCM degradation rate under DFUS could be quantified by defining a synergistic index (SI) according to Eq. (1) [53]:

(1) $$\mathrm{SI}=\frac{E_{\mathrm{DFUS}}}{E_{f_{1}}+E_{f_{2}}}$$

where *E* is any measurable effect such as pollutant degradation rate, and *f_1_* and *f_2_* are either of the input frequencies of the DFUS. Fig. 3 shows the PCM degradation SI versus the total combined calorimetric power applied under DFUS. For all the power combinations, an SI ranging from around 1.7 to 1.2 is recorded, suggesting that DFUS enhanced the degradation rate for all the studied cases, which agrees with literature investigating similar DFUS systems [53], [54]. However, increasing the total applied power decreases the SI forming a plateau, suggesting that operating the horn and plate transducer at the lowest and the highest effective powers respectively, is the most beneficial in terms of synergy.

**Fig. 4:**
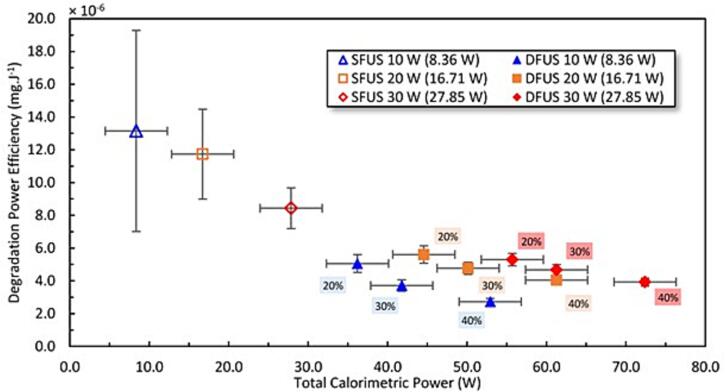
The degradation efficiency (the mass of degraded PCM mass per spent energy) versus the total calorimetric power exerted on the solution. The legend shows the driving power for the plate transducer where the corresponding calorimetric powers are mentioned in brackets. The percentage of the power amplitude of the horn is labelled next to the data.

The power efficiency of the PCM degradation is assessed using the degradation data and the calorimetric powers mentioned in Table 1 to calculate the mass of degraded PCM per spent energy (mg.J^−1^). This is then plotted as a function of total applied calorimetric power as presented in Fig. 4. Under SFUS (500 kHz), the efficiency decreases sharply with increasing power. The application of DFUS further decreases the efficiency, especially with increasing horn power. However, the drop under DFUS is not as sharp as that for SFUS. Also, unlike SFUS increasing the plate transducer power under DFUS resulted in a plateau, which implies a larger mass of PCM could be degraded at higher powers while maintaining the degradation efficiency. The comparison of the trends of SFUS and DFUS data suggests that the degradation of the pollutant is less limited under DFUS by the applied power i.e., DFUS could be more energy efficient than SFUS, despite the higher power consumption.

### 3.2 ROS dosimetry

**Fig. 5:**
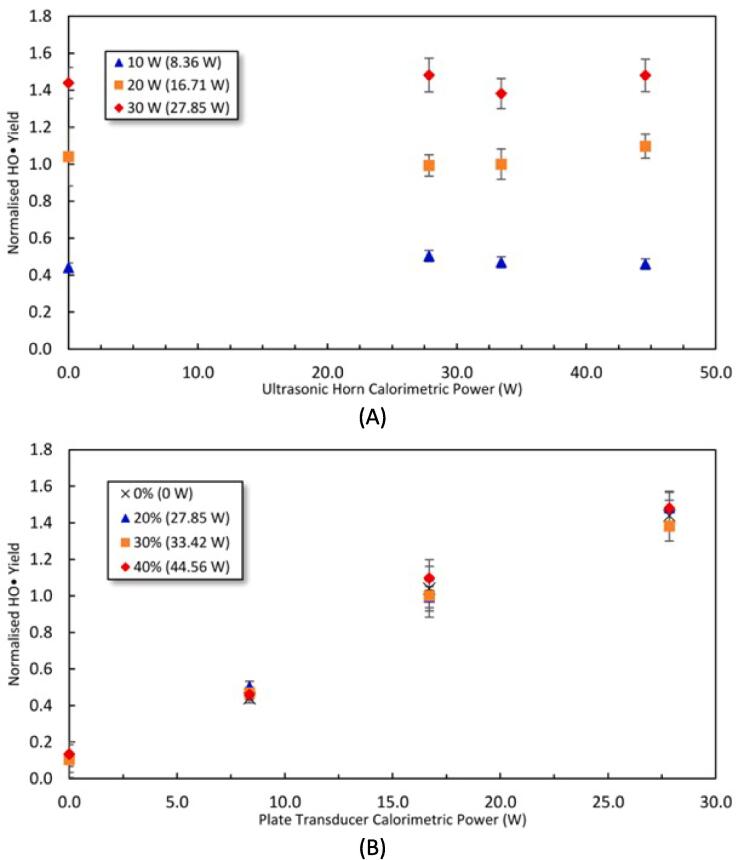
Normalised HO• yield as a function of increasing the driving power of A) the ultrasonic horn, and B) the plate transducer. The results presented at 0 W in the figures are for the SFUS of either of the frequencies in the absence of the other frequency. The legend shows the driving power for the plate transducer in (A) and the horn in (B) where the corresponding calorimetric powers are mentioned in brackets. The HO• yields have been normalised against the yield of 1.252±0.080 obtained under DFUS (500 kHz at 20 W and 30% for the 20 kHz horn).

Fig. 5 shows the effect of the sonication power on the yield of HO•, which is considered the main oxidant for degrading PCM [42]. The results presented at 0 W in the figure are for the SFUS. Fig. 5(A) reveals that applying DFUS as well as varying the power of the horn has very little impact on the yield of HO•. In contrast, Fig. 5(B) shows the yield of HO• increases almost linearly with increasing the power of the plate transducer, which has been reported for SFUS [12], [55], [56], [57], [58]. Similarly, the slight drop in the increase rate of the HO• yield at high powers (observed in Fig. 5B) has been reported previously for SFUS [55], [57], [58] and was attributed to attenuation of the sound field by the formation of larger bubbles at higher powers [59]. Excessive acoustic powers have also been shown to push cavitation bubbles away from the pressure antinode [60], which would also reduce cavitation activity, hence the yield of HO•.

The impact of the sonication power on the yield of the total ROS (HO• + H_2_O_2_) (shown in Fig. S2 of the supplementary data) was found to be similar, meaning that the power of the horn does not noticeably vary the yield of H_2_O_2_ as well.

### 3.3 Sono- and sonochemi-luminescence intensity

#### 3.3.1 Sonoluminescence intensity

**Fig. 6:**
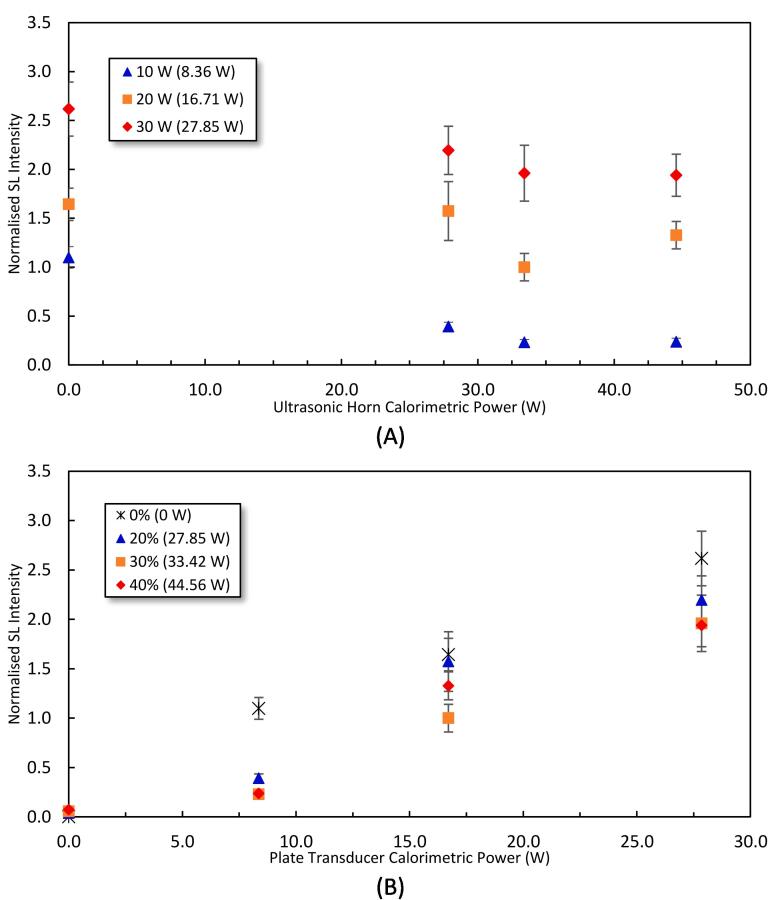
Normalised SL intensity as a function of the calorimetric power of A) the ultrasonic horn, and B) the plate transducer. The legend shows the driving power for the plate transducer in (A) and the horn in (B) where the corresponding calorimetric powers are mentioned in brackets. The SL intensities have been normalised against the SL intensity of 2.26×10^7^±2.23×10^6^ obtained under DFUS (500 kHz at 20 W and 30% for the 20 kHz horn).

**Fig. 7:**
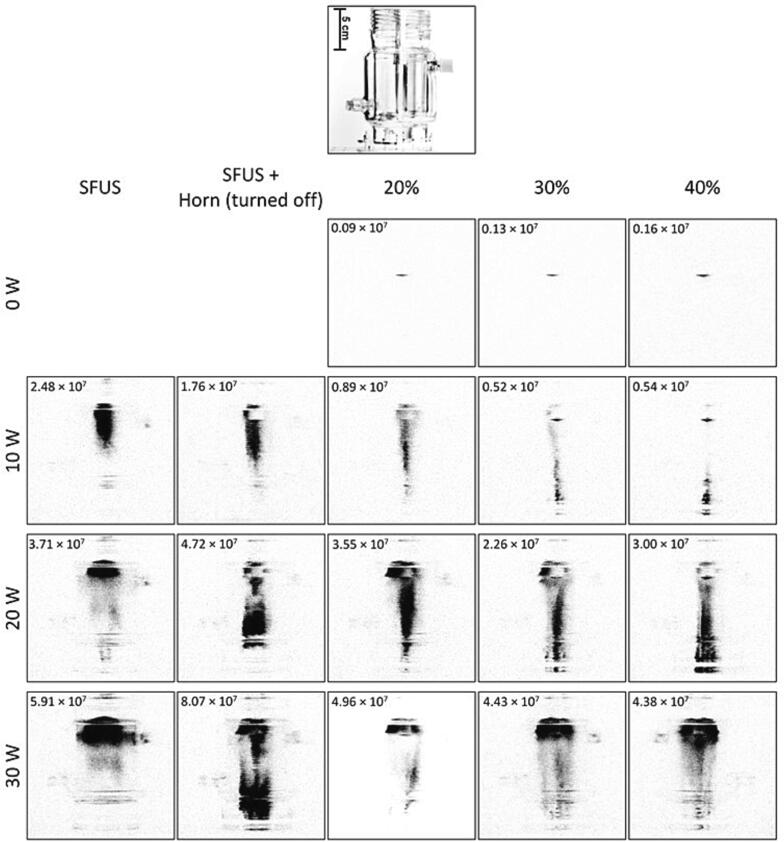
The spatial distribution of SL for the studied power combinations of the plate transducer and the Horn under SFUS and DFUS. For better visibility, the images are inverted, and the black spots represent the SL. The average overall SL intensity recorded for each power combination is mentioned in the corresponding picture. The picture of the empty reactor is presented as a guide/scale.

Fig. 6 shows the normalised overall SL intensity as a function of the horn (Fig. 6A) and plate transducer (Fig. 6B) calorimetric powers and shown in Fig. 7 are the spatial distribution of the SL emitting cavitation bubbles in the reactor for the studied driving power combinations. The effect of the physical body of the horn (no power) can act as a reflector to enhance the standing wave field [61] and increase the SL intensity, which is what is observed at the 20 and 30 W (Fig. 7). In contrast, turning the horn on and increasing the horn power from 20% to 30% decreases the overall SL intensities (Fig. 6A) as well as the SL spatial distribution (Fig. 7). This decrease in the SL intensity is attributed to the dispersion of the SL-emitting bubbles in the reactor, and the higher the driving power the closer the light-emitting zones are to the plate transducer at the bottom of the reactor. When the power of the horn is further increased from 30% to 40%, the overall SL increased slightly for plate transducer power of 10 and 20 W (Fig. 6A). Conversely, for 30 W transducer power, increasing horn power from 20% to 40% decreased the overall SL intensity (Fig. 6A) and the light-emitting zones are stronger closer to the liquid surface (Fig. 7).

Increasing plate transducer power increases the overall SL intensity (Fig. 6B), which is in accordance with the literature [61], [62], [63], with more SL-emitting bubbles near the liquid surface, which is caused by the attenuation of the acoustic field by large, coalesced bubbles [59], [64].

#### 3.3.2 Sonochemiluminescence intensity

**Fig. 8:**
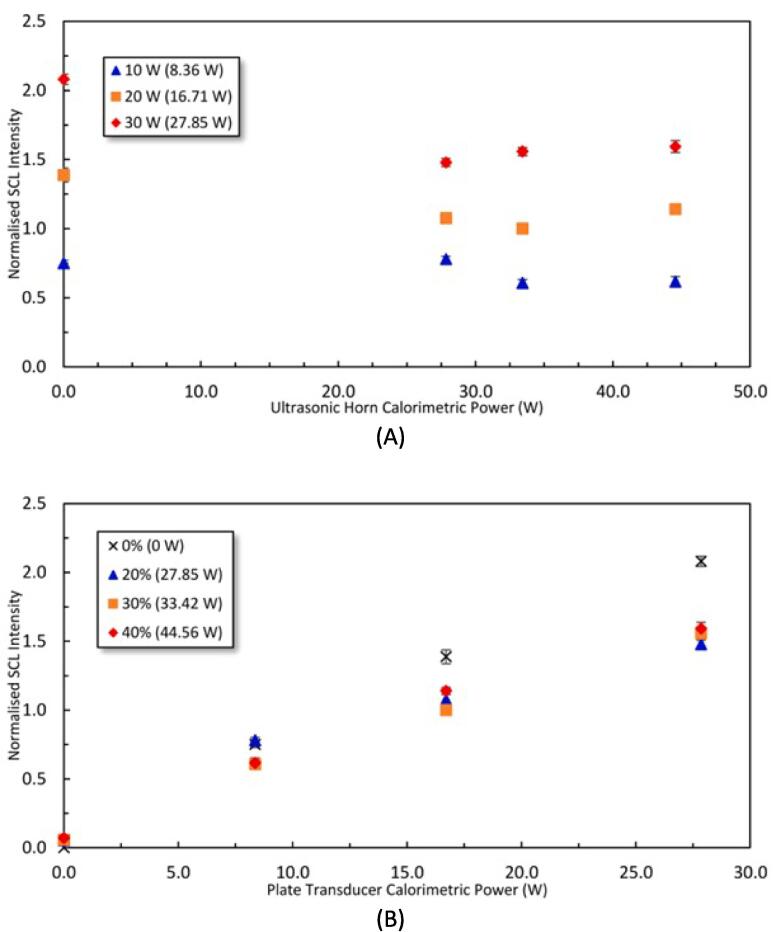
Normalised SCL intensity as a function of the calorimetric power of A) the ultrasonic horn, and B) the plate transducer. The legend shows the driving power for the plate transducer in (A) and the horn in (B) where the corresponding calorimetric powers are mentioned in brackets. The SCL intensities have been normalised against the SCL intensity of 3.92×10^7^±3.31×10^3^ obtained under DFUS (500 kHz at 20 W and 30% for the 20 kHz horn).

The effect of the sonication power on the overall SCL intensity is presented in Fig. 8 and shows a very similar trend as observed for SL (Fig. 6). There are however slight differences, that is the decrease in the SCL from SFUS to DFUS was more severe at higher powers of the plate transducer (Fig. 8A). Also, a further increase in the power of the horn does not further decrease the SCL as observed with SL, except for the plate transducer at the lowest power studied. In addition, for SL, increasing the driving power of the plate transducer caused the SFUS and DFUS curves to approach each other (Fig. 6B) whereas, for SCL, the SFUS and DFUS curves diverge with increasing plate transducer power (Fig. 8B).

**Fig. 9:**
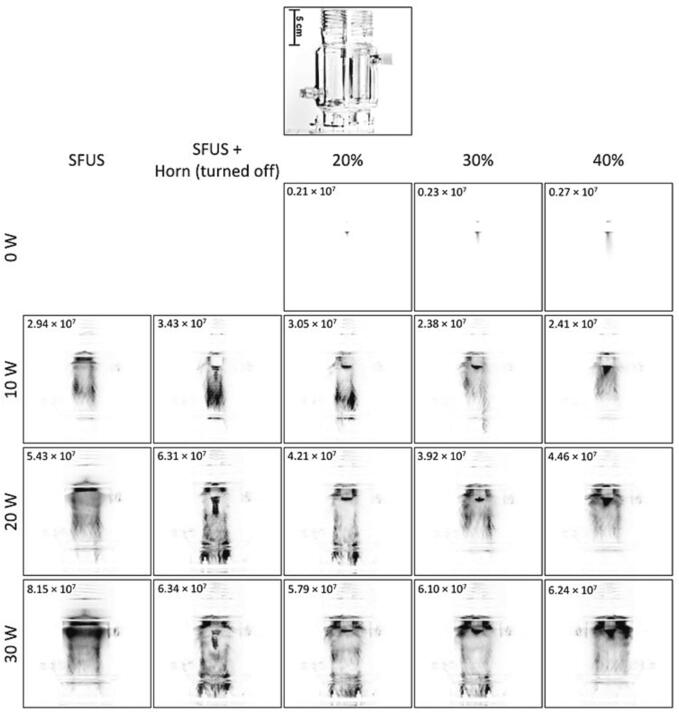
The spatial distribution of SCL for the studied power combinations of the plate transducer and the Horn under SFUS and DFUS. For better visibility the images are inverted, and the black spots represent the SCL. The average overall SCL intensity recorded for each power combination is mentioned in the corresponding picture. The picture of the empty reactor is presented as a guide/scale.

The effect of various driving power combinations on the spatial distribution of SCL is presented in Fig. 9. The results show that, unlike for the SL (Fig. 7), DFUS does not impact the spatial distribution of the SCL bubbles considerably, suggesting that DFUS has a larger impact on the stable cavitation bubbles emitting SL. Also, a comparison of the SL and SCL photographs for each driving power combination (Fig. 7 and Fig. 9) shows that to a major extent, the zones emitting SL and SCL differ, confirming different groups of bubbles contributing to SL and SCL [65], [66], [67], [68].

## 4 Discussions

The results in section 3.3 showed that applying DFUS did not result in any considerable enhancement in the sonochemical activity (ROS yields or SCL), and it decreased the overall SL intensity as well. However, DFUS remarkably enhanced the degradation rate of PCM and the accumulation of the UV-absorbing intermediates of sonodegradation. In addition, the plateau of the degradation efficiency under DFUS (Fig. 4) revealed that degradation of a higher mass of the pollutant is feasible under DFUS by increasing the power. Sonodegradation was associated with the oxidation of PCM molecules by HO•. Hence, the findings suggest that the enhancement in the degradation rate could be attributed to the higher availability of PCM molecules to react with HO•. Then, the correlations between PCM degradation rate, ROSs yields, SL and SCL are discussed to further clarify the mechanism through which the enhancement is achieved.

### 4.1 Correlation between major ROS

**Fig. 10:**
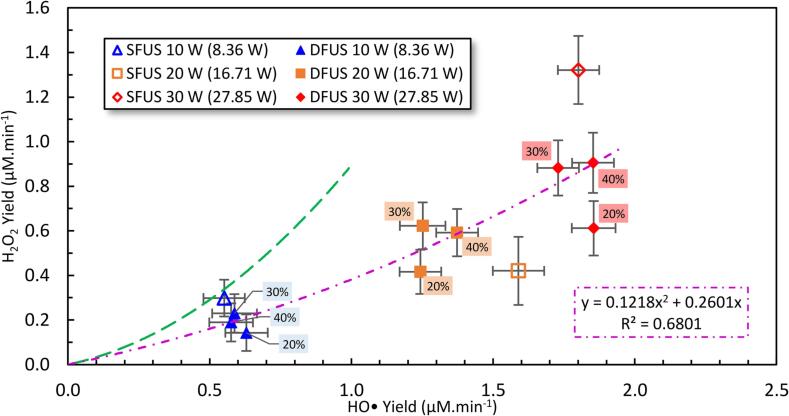
The yield of H_2_O_2_ as a function of HO• yield, measured at different sonication powers. The purple dash-dot line is fitted to guide the eye. The legend shows the driving power for the plate transducer where the corresponding calorimetric powers are mentioned in brackets. The percentage of the power amplitude of the horn is labelled next to the data. The previously [42] observed trends between the yield of H_2_O_2_ and HO• obtained for 22 – 2000 kHz under SFUS and DFUS are presented by green dashed lines.

Fig. 10 shows the correlation between the yield of H_2_O_2_ and HO• under different sonication powers. The green trendline represents the trend obtained under SFUS and DFUS from our previous study [42] on a range of 22 – 2000 kHz plate transducer frequencies at fixed driving power of 20 W (SFUS), paired with a 20 kHz horn ultrasonic horn with an amplitude of 20% of maximum (DFUS). In that study, it was concluded that the recombination of the HO• radicals produces H_2_O_2_, the yield of which increases parabolically (the green dashed line) with that of HO• (d[H_2_O_2_]/dt = k [HO•]^2^). Fig. 10 indicates that the power studies data points follow a distinct new parabolic trend with a lower profile (the purple dotted dash trendline). The lower trendline indicates that under the conditions presented here lower amounts of HO• combined to yield H_2_O_2_. Despite the previous study [42], the focus in the present study is only on the combination of a 500 kHz plate transducer with and without a 20 kHz horn in DFUS at different powers. Therefore, the new trend might be due to the specific effect the secondary frequency has on each primary frequency [40], [42]. The new trend also means more hydroxyl radicals could be available to degrade the pollutant, which has a stronger oxidative potential [42]. Nevertheless, the reason for this change in the recombination threshold of HO• is still unclear and further investigations are required for clarification.

### 4.2 Correlations between ROS Yield, SCL, and SL intensity

**Fig. 11:**
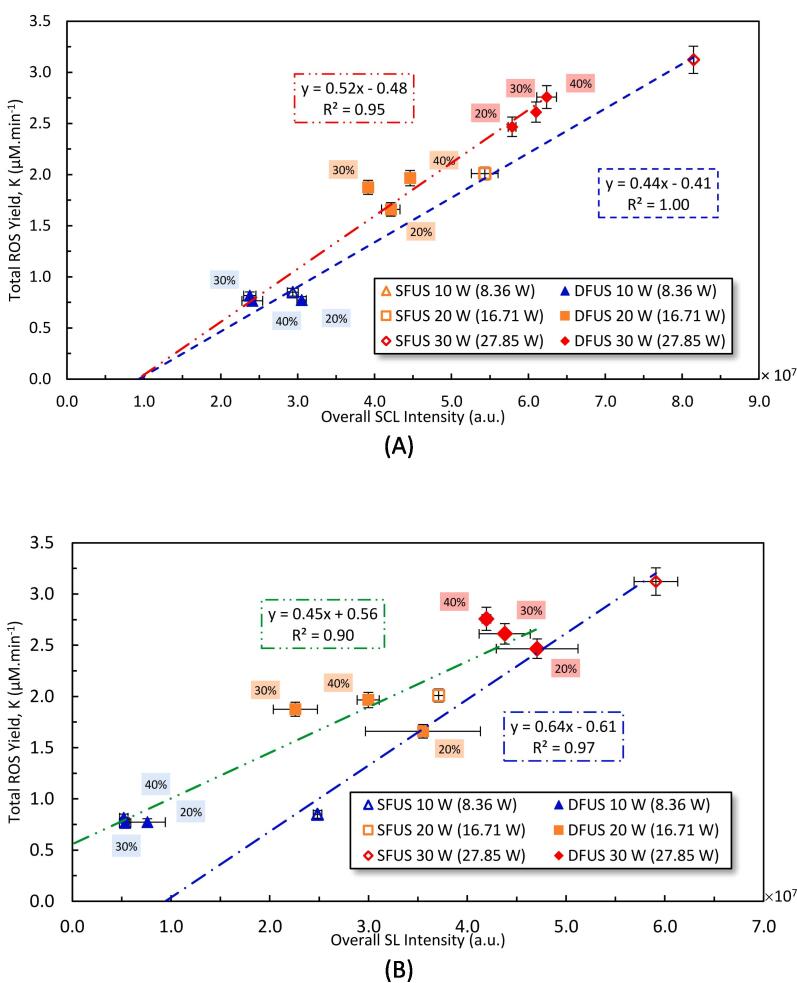

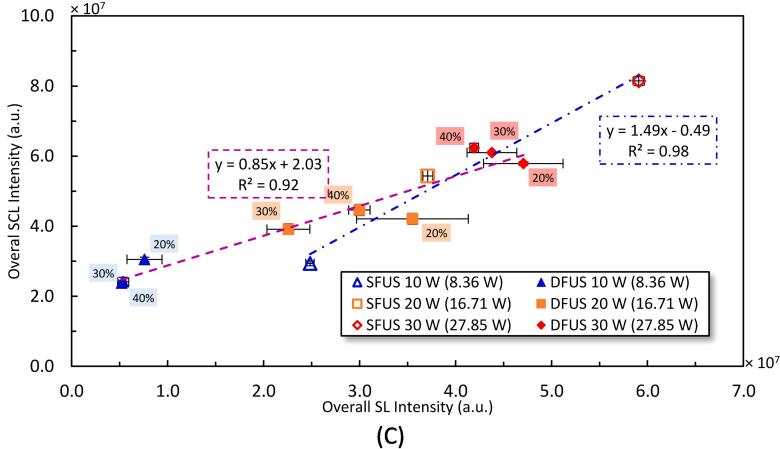
The yield of total ROS as a function of A) overall SCL intensity, B) overall SL intensity, and C) the overall SCL intensity versus the overall SL intensity, measured at different sonication powers. The legend shows the driving power for the plate transducer where the corresponding calorimetric powers are mentioned in brackets. The percentage of the power amplitude of the horn is labelled next to the data.

Plotted in Fig. 11 are the correlations between the total ROS yield and the overall SCL as well as SL intensities, measured at the studied power combinations. A strong correlation is observed between the total ROS and the overall SCL intensity (Fig. 11A). This is expected as the methods used to obtain total ROS and SCL are the respective KI dosimetry and luminol, both of which depend on the sonogenerated hydroxyl radicals. The negative intercept could be explained by the sensitivities of KI dosimetry and luminol. KI is less sensitive than luminol in reacting with the sonogenerated ROS [69] which causes less ROS to be detected than generated. The slightly higher slope obtained under DFUS compared to SFUS is interesting, and could be attributed to the enhancement in the reaction of KI with the reacting oxidant species under DFUS.

Fig. 11(B) shows linear trends between the total ROS yield and the overall SL intensities for SFUS (blue dotted-dashed line) and DFUS (green dotted-dashed line) with non-zero intercepts. Similar trends are shown in Fig. 11(C) between the overall SCL and SL intensities. The different linear relationships between SFUS and DFUS is attributed to the different bubble size distribution. It has been shown for SFUS [63] that SL bubbles are larger than SCL or ROS bubbles. Applying DFUS would make the bubbles smaller and decrease SL emission with increasing horn power as observed in Fig. 6(A). Therefore, the ratio of the ROS or SCL to SL bubbles would be lower under SFUS compared to DFUS. As the plate transducer power increases, it is likely the SL bubbles under DFUS are increased more than the ROS or SCL bubbles, giving rise to a smaller gradient compared to SFUS. This difference in the ratio of ROS or SCL to SL bubbles under DFUS and SFUS would explain the negative intercept for SFUS where SL is recorded when no ROS (or SCL) and vice versa for when ROS or SCL is observed when no SL is measured.

Carrying a normal distribution function (NDF) analysis [42] for the SL intensities of individual bubbles, which correlate with their size [15], [42], showed that DFUS makes the bubbles smaller as well as more uniform (Fig. S3 of the supplementary data). It is noteworthy that the NDF analysis did not show such a meaningful behaviour for the individual SCL bubbles (Fig. S3 of the supplementary data). An explanation is that the SCL intensity emitted by a cavitation bubble depends on its yield of ROS which is linked to the population of collapsing asymmetric or dancing bubbles rather than bubble size [67].

A common expectation from pairing the 20 kHz ultrasound with a high-frequency ultrasound (say 500 kHz) might be that the 20 kHz acoustic field grows the bubbles produced by the high-frequency ultrasound [28], thus larger bubbles and consequently higher overall SL intensities can be expected under DFUS. However, the impact of DFUS on the cavitation bubbles’ size and oscillation is more complicated [40], [70], [71]. Also, the results of previous studies [34], [42] showed that depending on the sonication condition under DFUS, the outcome might contradict the common expectation.

### 4.3 Correlations between PCM degradation and ROS Yield, SCL as well as SL intensity

**Fig. 12:**
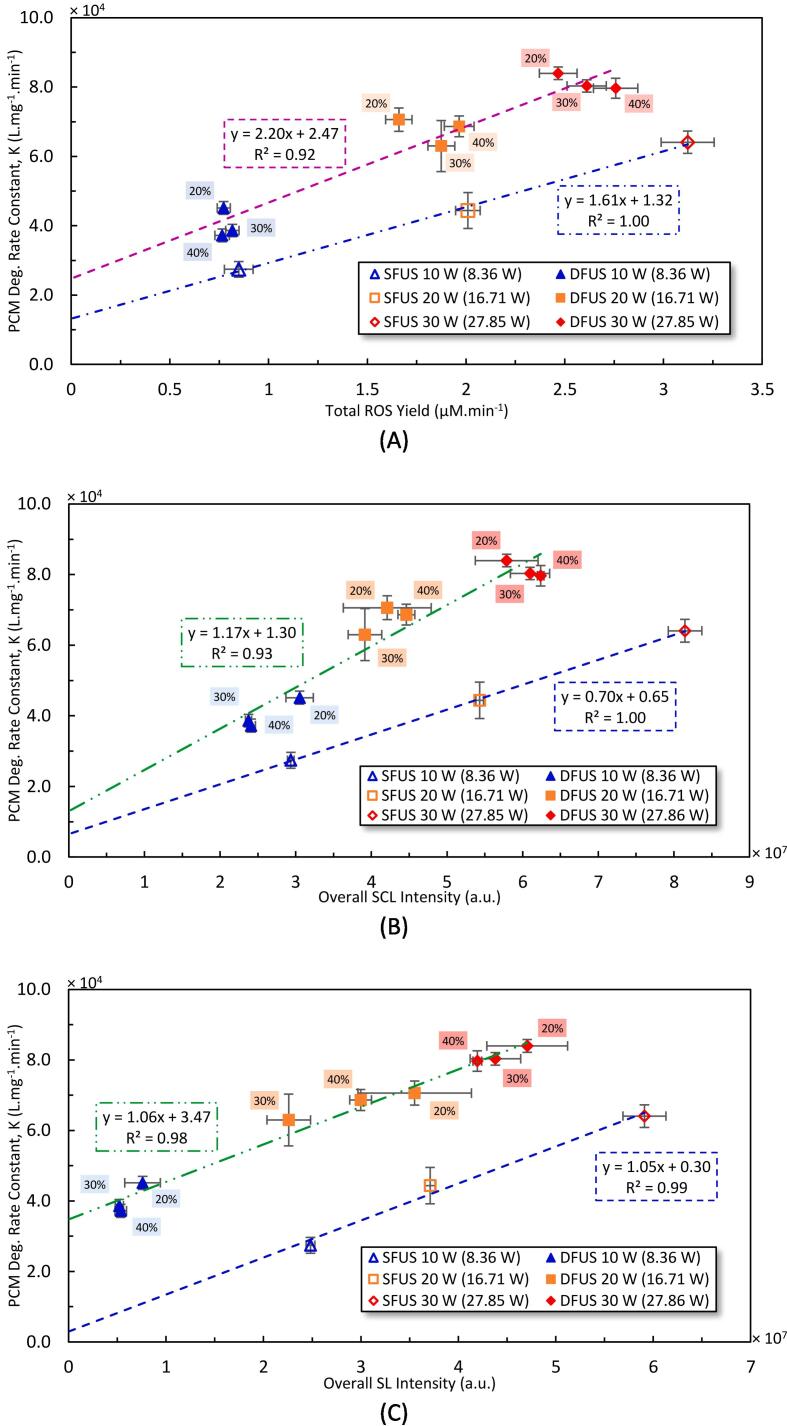
The relationship between the PCM degradation and A) yield of total ROS, B) the overall SCL intensity as well as C) the overall SL intensity, measured for the studied driving powers of the Horn and the plate transducer, under SFUS and DFUS. The legend shows the driving power for the plate transducer where the corresponding calorimetric powers are mentioned in brackets. The percentage of the power amplitude of the horn is labelled next to the data.

The correlation between the degradation rate constant of PCM and the total ROS yield and overall SCL intensity are shown in Fig. 12(A and B), respectively. Two distinct linear correlations can be found for SFUS and DFUS, both with a positive intercept, which could be attributed to the insensitivity of KI dosimetry as discussed above (larger intercept for total ROS in Fig. 12 A than SCL Fig. 12B). Also, at the concentrations used, PCM (log P = 0.48) [72], [73] is less hydrophilic compared to KI and luminol (log P = − 0.19) [74] and therefore, PCM would be closer to the bubble surface compared to KI and luminol. This would explain the positive intercept where PCM degradation is detected when no ROS or SCL is detected.

The strong linear correlations indicate that the main degradation mechanism of PCM under SFUS and DFUS is oxidation by the sono-generated ROS. However, the trendline of DFUS has almost double the slope of that of SFUS. The slope could be interpreted as the efficiency of the degradation reaction for a given sonochemical activity. Under the conditions applied in this study, PCM is a hydrophilic compound [73] that lies more in the bulk solution rather than near the surface of the cavitation bubbles [75]. On the other hand, sonodegradation is a heterogenous reaction [76] that relies on the transport of the reactant molecules towards the reaction site i.e., PCM towards the surface of the cavitation bubbles. As discussed previously (Fig. 5, as well as Fig. S2 of the supplementary data), applying DFUS does not noticeably affect the yield of ROS. The NDF analysis has shown that DFUS makes the SL bubbles smaller and more distributed throughout the reactor (Fig. 7), therefore causing the observed decrease in the overall SL intensity but no significant impact on the SCL bubble size distribution (Fig. S3 of the supplementary data). It is likely that the more homogenous distribution of SL bubbles and the additional agitation introduced to the solution by the ultrasonic horn may have further increased the availability of PCM near the bubble liquid interface (reaction site). Therefore, the improvement in the degradation rate under DFUS could be due to the improvement in the mass transfer of the pollutant from the bulk solution toward the cavitation bubbles.

Fig. 12(C) is a plot of the PCM degradation rate constant versus the overall SL intensities showing two distinct linear correlations for SFUS and DFUS, with the same gradient and positive intercepts that differ by an order of magnitude. For DFUS, the intercepts imply that the degradation threshold is lower than that of the SL emission i.e., degradation starts before any SL could be detected. One may assume pyrolysis as the degradation mechanism due to the linearity shown in Fig. 12(C). However, if this was the case, higher degradation rates could be expected under SFUS where higher overall SL intensity (higher collapse intensity) was measured. In addition, under the experimental conditions used, it is unlikely that a hydrophilic pollutant like PCM with such low volatility (vapour pressure of 6.29×10^−5^ mm Hg at 25 °C) [77] can evaporate into the bubbles’ core. Even for volatile organic compounds such as phenol and levofloxacin, oxidation by HO• has been reported as the main sonodegradation mechanism [45], [78], [79], [80]. The only reason for the linear trends observed in Fig. 12(C) is the linear correlation of the PCM degradation with overall SCL intensities as well as the linear correlations found between the overall SL and SCL intensities (α∝βandβ∝γthereforeα∝γ). Nevertheless, though minimal, there is a non-zero probability of pyrolysis occurring through nano-droplet injection, or thermolysis by the supercritical water film.

## 5 Conclusions

Although DFUS under the studied condition had almost no effect on the yield of ROS, it considerably improved the overall degradation rate of PCM, leading to a synergistic effect at all the studied driving powers (with SI ranging from 1.2 to 1.7). However, varying the power of the horn was shown to have no significant effect either on the PCM degradation rate or on the ROS yield, suggesting that there is a threshold power for the horn above which the improvement is achieved. The results of the current study suggest that even the minimum applied horn power (20%) is above the threshold. Therefore, further studies are required to determine the threshold power as well as to clarify the impact of other parameters such as horn depth, the frequency of the other applied ultrasound or the degree of the hydrophilicity of the target pollutant on it. The SL and SCL measurements as well as the normal distribution analyses revealed that DFUS mainly affected SL cavitation bubbles, by making them smaller and more uniform in size. A comparison of the PCM degradation rate versus sonochemistry indicators such as SCL revealed that DFUS enhanced the efficiency of the degradation for the same sonochemical activity, in comparison to SFUS. This is attributed to the enhancement in the mass transfer of the PCM to the cavitation bubble under DFUS. The advantage of DFUS over SFUS is particularly important where the target pollutant is highly toxic and hydrophilic, and the degradation rate is diffusion controlled.

## CRediT authorship contribution statement

**Mehrdad Zare:** Conceptualization, Methodology, Investigation, Writing – original draft, Writing – review & editing, Visualization. **Madeleine J. Bussemaker:** Writing – review & editing. **Efraím A. Serna-Galvis:** Conceptualization, Writing – review & editing, Visualization. **Ricardo A. Torres-Palma:** Writing – review & editing, Supervision, Funding acquisition. **Judy Lee:** Conceptualization, Methodology, Resources, Writing – review & editing, Visualization, Supervision, Project administration, Funding acquisition.

## Declaration of Competing Interest

The authors declare that they have no known competing financial interests or personal relationships that could have appeared to influence the work reported in this paper.

## Data Availability

Data will be made available on request.
