# Supplementary material for: Impact of sonication power on the degradation of paracetamol under single- and dual-frequency ultrasound
Source: Ultrason Sonochem. 2023 Aug 19;99:106564. doi: 10.1016/j.ultsonch.2023.106564 (PMC10474498; doi:10.1016/j.ultsonch.2023.106564)
Supplement: Supplementary Data 1 [file mmc1.docx]

**SUPPLEMENTARY DATA
Impact of Sonication Power on the Degradation of Paracetamol under Single- and Dual-Frequency Ultrasound**

Mehrdad Zare^a^, Madeleine J. Bussemaker^a^, Efraím A. Serna-Galvis ^b,c^, Ricardo A. Torres-Palma^b^, Judy Lee^a^

^a^ School of Chemistry and Chemical Engineering

University of Surrey, Guildford, GU2 7XH, United Kingdom

^b^ Grupo de Investigación en Remediación Ambiental y Biocatálisis (GIRAB), Instituto de Química, Facultad de Ciencias Exactas y Naturales, Universidad de Antioquia UdeA, Calle 70 # 52-21, Medellín, Colombia

^c^ Catalizadores y Adsorbentes (CATALAD), Instituto de Química, Facultad de Ciencias Exactas y Naturales, Universidad de Antioquia UdeA, Calle 70 # 52-21, Medellín, Colombia

# Confirmation of the linearity of UV-Visible spectrophotometry and liquid chromatography (LC)-mass spectrometry (MC) results

To confirm the reliability of the UV-Visible spectrophotometer (UV-Vis.) the linearity of the methods was investigated using standard solutions with concentrations ranging from 0.01 to 100 mg.L^-1^. Fig-S. 1 shows the results of the investigation. The results showed that the measurement range of this method is 0.5 to 50 mg.L^-1^.

Fig-S. 1 – The results of the linearity test, for the UV-Vis. method for measurement of PCM concentration. The linear fit and the corresponding equation and R^2^ are presented for the linear section of the curve.

# The comparison of the pseudo-1^st^- and pseudo-2^nd^-order reaction rate models for the PCM degradation data

The statistical comparison of the pseudo-1^st^- and pseudo-2^nd^-order reaction kinetic models are presented in Table-S. 1. Although the coefficient of determination (R^2^) is high for both models, the sum of squared error (SSE) for the pseudo-2^nd^-order model is at least one order of magnitude smaller than that of the pseudo-1^st^-order model. This shows the better compliance of the pseudo-2^nd^-order model to the PCM degradation data.

Table-S. 1 – The statistical comparison of the pseudo-1^st^- and the pseudo-2^nd^-order reaction kinetic models in terms of the coefficient of determination (R^2^) and the sum of squared errors (SSE).

| Power Combination |  | Pseudo-1^st^-Order | | |  | Pseudo-2^nd^-Order | | |
| --- | --- | --- | --- | --- | --- | --- | --- | --- |
|  |  | K × 10 ^3^  (min^-1^) | R^2^ | SSE × 10 ^5^ |  | K × 10 ^4^  (L.mg^-1^.min^-1^) | R^2^ | SSE × 10 ^6^ |
| 10 W |  | 1.46 | 0.998 | 4.58 |  | 3.02 | 0.994 | 1.52 |
| 20 W |  | 2.71 | 0.995 | 34.0 |  | 5.47 | 0.991 | 7.53 |
| 30 W |  | 3.50 | 0.988 | 130 |  | 7.05 | 0.978 | 31.5 |
| 10 W 20% |  | 2.40 | 0.999 | 6.43 |  | 4.97 | 0.999 | 0.576 |
| 10 W 30% |  | 1.92 | 0.998 | 5.48 |  | 4.25 | 0.996 | 1.94 |
| 10 W 40% |  | 1.78 | 0.995 | 14.7 |  | 4.09 | 0.991 | 4.28 |
| 20 W 20% |  | 3.44 | 0.999 | 7.69 |  | 7.76 | 0.999 | 2.52 |
| 20 W 30% |  | 3.14 | 0.999 | 8.95 |  | 6.30 | 0.998 | 3.10 |
| 20 W 40% |  | 3.38 | 0.999 | 5.33 |  | 7.55 | 0.999 | 1.32 |
| 30 W 20% |  | 4.17 | 0.998 | 27.9 |  | 9.24 | 0.998 | 4.84 |
| 30 W 30% |  | 4.03 | 0.998 | 23.7 |  | 8.83 | 0.998 | 4.11 |
| 30 W 40% |  | 4.00 | 0.998 | 24.8 |  | 8.76 | 0.999 | 3.04 |

# The raw experimental data of 500 kHz under DFUS at 20 W 30%

Table-S. 2 presents the raw data experiment results for 500 kHz under DFUS at the power combination of 20 W and 30%, based on which the corresponding data of other studied power combinations presented in the article were normalised.

Table-S. 2 – The raw experimental data of 500 kHz under DFUS at the power combination of 20 W 30%

| **No.** | **Parameter** | **Unit** | **Value** |
| --- | --- | --- | --- |
| 1 | Degradation Rate Constant, K | L.mg^-1^.min^-1^ | 6.30 × 10 ^-4^ ± 7.34 × 10 ^-5^ |
| 2 | HO• Yield | μM.min^-1^ | 1.252 ± 0.080 |
| 3 | SL Intensity | a.u. | 2.26 × 10 ^7^ ± 2.23 × 10 ^6^ |
| 4 | SCL Intensity | a.u. | 3.92 × 10 ^7^ ± 3.31 × 10 ^3^ |

# The impact of the sonication power on the yield of the total ROS (HO• + H_2_O_2_)


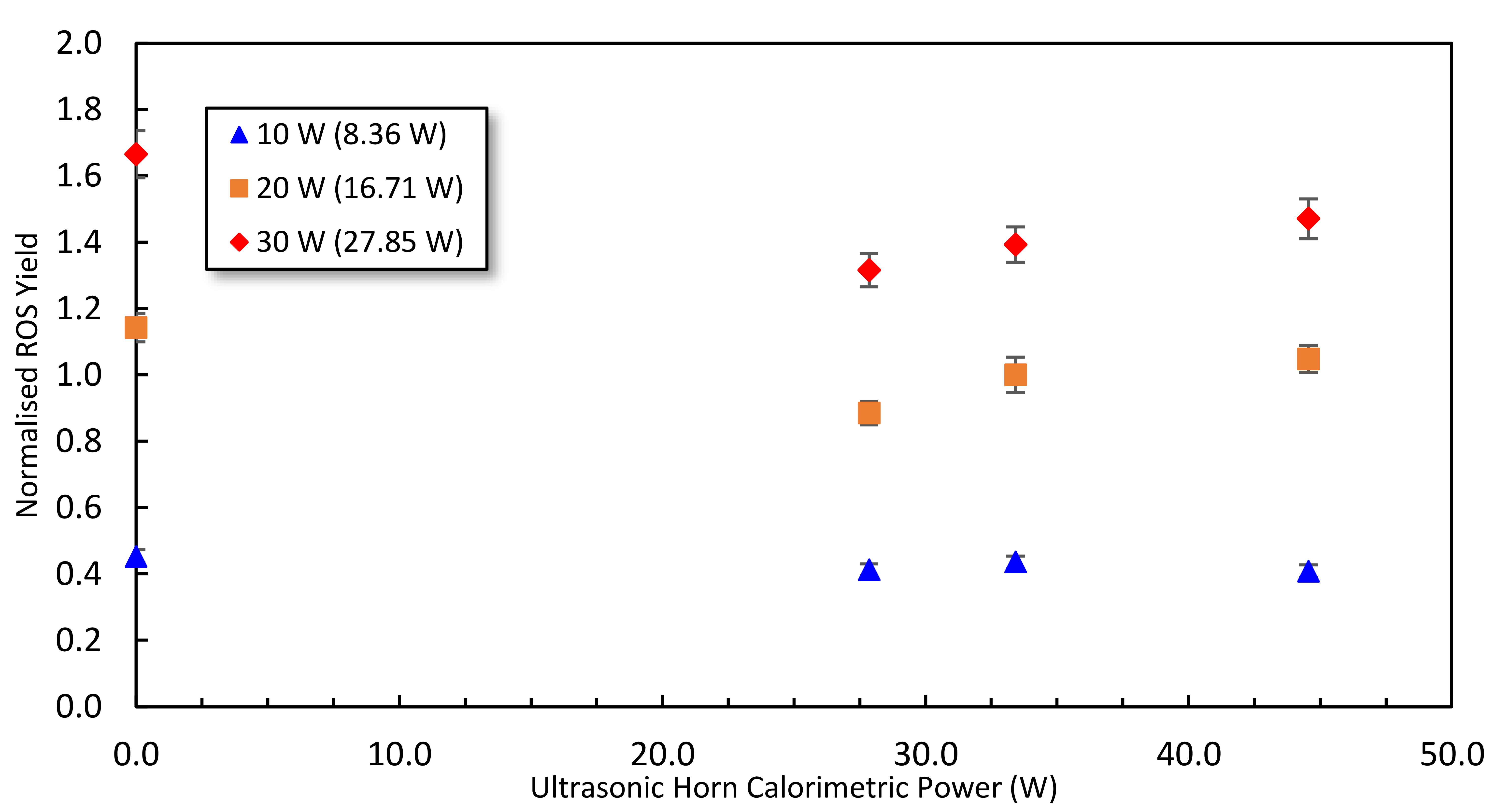


(A)


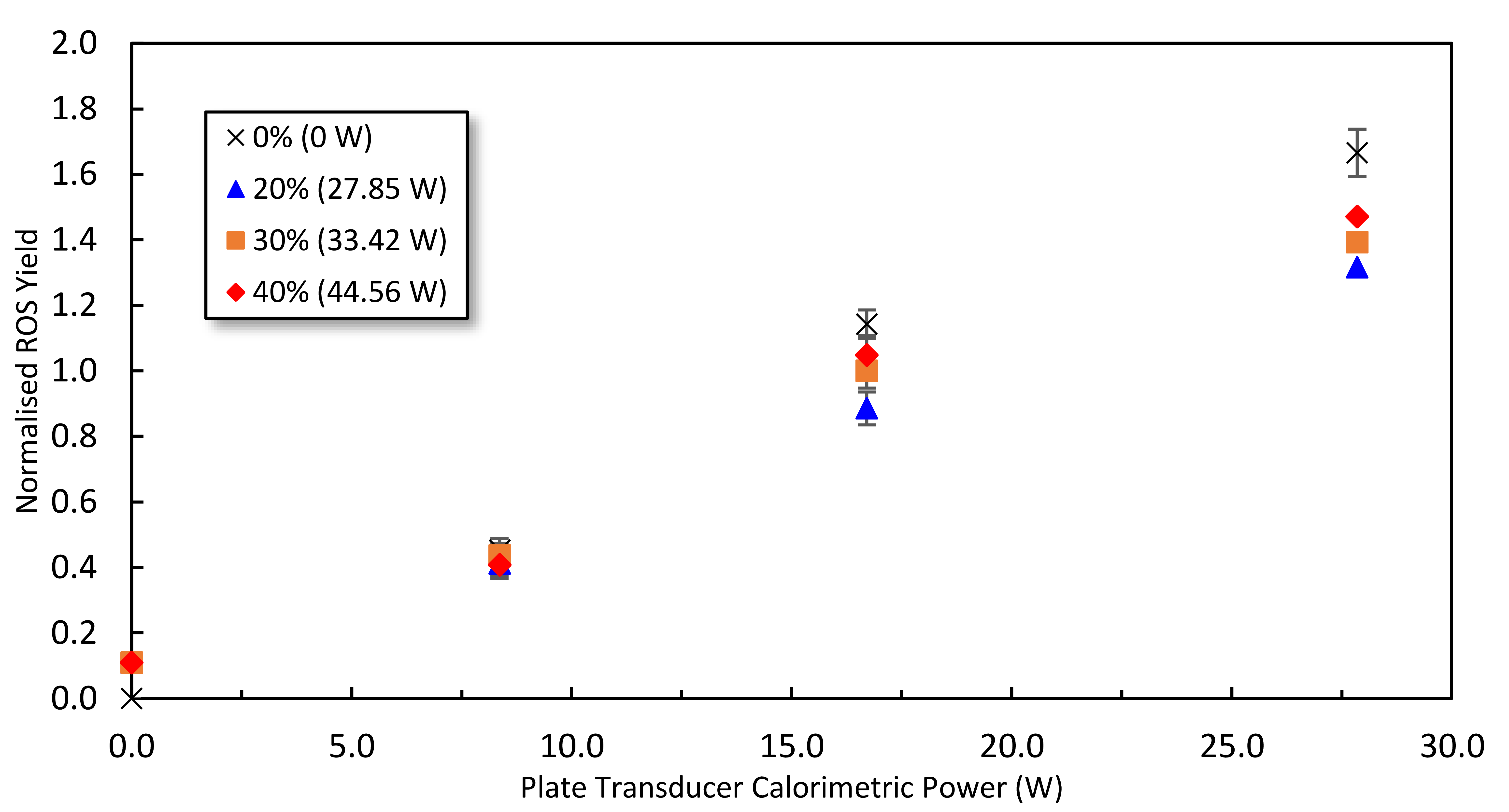


(B)

Fig-S. 2 – Normalised total ROS yield as a function of increasing driving power of A) the ultrasonic horn, and B) the plate transducer. The legend shows the driving power for the plate transducer in (A) and the horn in (B) where the corresponding calorimetric powers are mentioned in brackets. The total ROS yields have been normalised against the yield of 1.874 ± 0.068 obtained under DFUS (500 kHz at 20W and 30% for the 20kHz horn).

# Probability Density Analysis for SL/SCL Intensities

Fig-S. 2 presents the normal distribution function (NDF) for the individual bubble’s SL intensities recorded by each camera pixel, for all the studied power conditions. For an NDF curve, the peak occurs at the average of the studied values, and the width of the peak is directly proportional to the standard deviation of the data. In other words, the peak and its width represent the average as well as the degree of uniformity of the studied data, in this case, the individual bubble’s SL and the corresponding bubble diameters. Accordingly, for all the studied power combinations, applying DFUS makes the SL emitting bubbles smaller and more uniform.





(A)





(B)





(C)

Fig-S. 2 – The effect of the 20 kHz ultrasound on the normal distribution of the individual bubble’s SL intensities (bubble diameters) under the driving powers(calorimetric power) of A) 10 W (8.36 W), B) 20 W (16.71 W), and C) 30 W (27.85 W), applied to the plate transducer (500 kHz).

Also, a comparison of the DFUS data in Fig. 10 (A) of the manuscript with the corresponding curves in Fig-S. 2 reveals that the smaller and the more uniform the bubbles are under DFUS of a certain power combination, the closer the corresponding DFUS data point is to the linear trend (the red dashed line). In other words, the smaller DFUS makes the cavitation bubbles the more it pushes the overall SL intensity to a linear correlation with the yield of ROS.

Fig-S. 3 shows the same NDF analysis for the individual SCL intensities. A comparison of Fig-S. 3 with Fig. 10 (A) reveals that there is no such a strong relationship, as discussed above, between the NDF of individual SCLs and where the data point lies in Fig. 10 (A). An explanation is that the SCL intensity emitted by a cavitation bubble is dependent on the yield of HO• generated by the bubble rather than its size.





(A)





(B)





(C)

Fig-S. 3 – The effect of the 20 kHz ultrasound on the normal distribution of the individual bubble’s SCL intensities (bubble diameters) under the driving powers(calorimetric power) of A) 10 W (8.36 W), B) 20 W (16.71 W), and C) 30 W (27.85 W), applied to the plate transducer (500 kHz).
